# The Antibody Genetics of Multiple Sclerosis: Comparing Next-Generation Sequencing to Sanger Sequencing

**DOI:** 10.3389/fneur.2014.00166

**Published:** 2014-09-16

**Authors:** William H. Rounds, Ann J. Ligocki, Mikhail K. Levin, Benjamin M. Greenberg, Douglas W. Bigwood, Eric M. Eastman, Lindsay G. Cowell, Nancy L. Monson

**Affiliations:** ^1^Department of Neurology and Neurotherapeutics, University of Texas Southwestern Medical Center, Dallas, TX, USA; ^2^Department of Clinical Sciences, University of Texas Southwestern Medical Center, Dallas, TX, USA; ^3^DioGenix Inc., Bethesda, MD, USA; ^4^Department of Immunology, University of Texas Southwestern Medical Center, Dallas, TX, USA

**Keywords:** multiple sclerosis, B cell, antibody, Roche 454, next-generation sequencing

## Abstract

We previously identified a distinct mutation pattern in the antibody genes of B cells isolated from cerebrospinal fluid (CSF) that can identify patients who have relapsing-remitting multiple sclerosis (RRMS) and patients with clinically isolated syndromes who will convert to RRMS. This antibody gene signature (AGS) was developed using Sanger sequencing of single B cells. While potentially helpful to patients, Sanger sequencing is not an assay that can be practically deployed in clinical settings. In order to provide AGS evaluations to patients as part of their diagnostic workup, we developed protocols to generate AGS scores using next-generation DNA sequencing (NGS) on CSF-derived cell pellets without the need to isolate single cells. This approach has the potential to increase the coverage of the B-cell population being analyzed, reduce the time needed to generate AGS scores, and may improve the overall performance of the AGS approach as a diagnostic test in the future. However, no investigations have focused on whether NGS-based repertoires will properly reflect antibody gene frequencies and somatic hypermutation patterns defined by Sanger sequencing. To address this issue, we isolated paired CSF samples from eight patients who either had MS or were at risk to develop MS. Here, we present data that antibody gene frequencies and somatic hypermutation patterns are similar in Sanger and NGS-based antibody repertoires from these paired CSF samples. In addition, AGS scores derived from the NGS database correctly identified the patients who initially had or subsequently converted to RRMS, with precision similar to that of the Sanger sequencing approach. Further investigation of the utility of the AGS in predicting conversion to MS using NGS-derived antibody repertoires in a larger cohort of patients is warranted.

## Introduction

Diagnosing diseases that affect the central nervous system (CNS) is inherently challenging. Multiple sclerosis (MS) is an autoimmune-mediated disease that exemplifies this challenge since clinicians must use multiple diagnostic tools to obtain the required evidence of dissemination of disease separated in time and space according to the current McDonald criteria (1). This includes radiological tests that detect lesions in the brain and spinal cord by magnetic resonance imaging (MRI) and is supported by biological tests that detect a unique pattern of oligoclonal banding (OCB) in the cerebrospinal fluid (CSF).

Due to the complexity associated with the current standard of care for MS diagnosis, patients who suffer an initial acute onset of “MS-like” symptoms [referred to as a clinically isolated syndrome (CIS)] often have to wait before a diagnosis of MS is confirmed and treatment is initiated (2). Steps to shorten this time frame are an urgent matter in the field, considering that patients have a better prognosis if treated early (3). Radiological testing (i.e., MRI) has been instrumental in the diagnosis of MS, but the most frequently used biological test that supports MS diagnosis is the OCB test, which has relatively low diagnostic specificity when comparing test performance for MS vs. other neuro-inflammatory diseases (about 61%) (4–6).

The standardization of the OCB test to support an MS diagnosis led many neuroimmunologists in the field to focus on determining the role of B cells and their antibodies on the pathogenesis of MS (7–11). Early work by our group and others demonstrated that CSF-derived B cells from MS patients and CIS patients that convert to MS undergo extensive clonal expansion, skewing toward heavy chains of the fourth family, and accumulate somatic hypermutations (SHM) at an advanced rate (12–14). These features of antibody genetics are suggestive of a hyper-response to CNS antigens, but the targets of these CSF-derived B cells from MS patients remain elusive (15). More recently, however, our laboratory has discovered that the fourth family of heavy-chain antibody genes of CSF-derived B cells from MS patients accumulates replacement mutations at six codon positions more frequently than patients with other neurological diseases (OND) (16). B cells in MS lesions also display this pattern (17).

Using a custom algorithm to indicate the extent of mutation accumulation at these six codons in antibody gene repertoires, we developed a new biological test called the antibody gene signature (AGS), which demonstrated promise in a small pilot cohort in identifying patients who had one demyelinating event and who would convert to MS (16). However, these initial studies on the utility of AGS were based on Sanger sequencing, which is too laborious and expensive for routine use if this technology is developed as a clinical diagnostic test for MS in the future.

Next-generation DNA sequencing (NGS) might potentially provide a useful alternative in acquiring antibody gene repertoires to use for AGS calculations and is becoming routine in the field as evidenced by its commercial availability as a fee for service (Life Technologies, Illumina, and Seqwright among many others). The most common application of NGS to antibody genetics has focused on VDJ recombination gene selection for the purpose of analyzing lymphocyte clonality (18–21), and is now being utilized in the MS field (22). Since gene and SHM distributions are at the core of antibody genetics analysis (as well as AGS scoring), careful scrutiny of this platform and its ability to properly represent the antibody gene repertoire is warranted.

Our primary goal was to provide confirmation that the antibody gene repertoires generated by NGS would sufficiently represent the CSF-derived B-cell pool from MS patients. The data presented here demonstrate for the first time that antibody gene repertoires from individual CSF-derived B cells from the CSF of MS patients and those at high risk to convert, generated by the gold standard Sanger method, are reliably reflected in NGS-generated antibody gene repertoires from paired CSF-derived B-cell pools of the same patients. Furthermore, we confirmed that AGS scoring, generated using a high-throughput NGS approach of pooled CSF cells, also identified MS patients and those that would convert to MS with the same accuracy as AGS scoring using Sanger DNA sequencing of individual CSF B cells. This NGS approach provides a new method for measuring the biological changes observed in MS patients and demonstrates its potential as a diagnostic tool.

## Materials and Methods

### MS/CIS patient description and CSF sample preparation

Cerebrospinal fluid B cells from six CIS and two relapsing-remitting multiple sclerosis (RRMS) patients were used for this paired analysis. All CSF samples were collected in accordance with a protocol approved by the UT Southwestern Medical Center (UTSWMC) Institutional Review Board (IRB). CSF samples selected for comparative analysis were collected from eight patients who were either diagnosed with RRMS or CIS at the time of collection or who were subsequently diagnosed with RRMS (Table 1). Single CD19+ B cells were sorted into individual wells of a 96-well microtiter plate for single-cell Sanger DNA sequencing. At the same time, a pool of sorted CD19+ B cells from each patient was collected for NGS analysis. One pooled B-cell sample (C8) did not produce a detectable PCR product after nested PCR and thus was removed from the NGS cohort.

**Table 1:** **Patient sample summary**.

Patient ID	Initial diagnosis^a^	OCB status	Comments	Follow-up diagnosis^b^	Follow-up time^c^	Age^d^	Gender	Sanger AGS	NGS AGS
C1	CIS	NEG	High risk of RRMS	CIS	44	45	F	6.43	13.32
C2	CIS	POS	Single lesion^e^	CIS	26	34	F	13.07	4.43
C3	CIS	POS		RRMS	1	39	F	10.47	13.88
C4	CIS	POS	High risk of RRMS	RRMS	8	27	F	17.90	17.55
C5	RRMS	POS	On steroids	RRMS	36	19	F	16.73	8.21
C6	RRMS	POS		RRMS	25	19	F	17.62	10.26
C7	CIS	POS	High risk of RRMS	RRMS	31	33	M	22.26	18.01
C8	CIS	POS	Low risk of RRMS	RRMS	8	34	F	10.17	NA

*Initial diagnosis at the time of sample collection is indicated for each patient in the study*.

*OCB, oligoclonal bands; AGS, antibody gene signature; CIS, clinically isolated syndrome; RRMS, relapsing-remitting multiple sclerosis*.

*^a^At time of sampling using 2005 McDonald criteria*.

*^b^Using 2010 McDonald criteria*.

*^c^Since sampling (months)*.

*^d^At time of sampling (years)*.

*^e^By MRI of the brain*.

### Next-generation sequencing controls

Naïve (CD19+ CD27−) and memory (CD19+ CD27+) peripheral blood B-cell pools were isolated from three healthy control samples and used as process controls to evaluate batch to batch variation and to aid in the evaluation of potential sequence errors generated during processing. Peripheral blood from healthy control donors was collected in blood tubes containing heparin as an anti-coagulant (BD, Franklin Lakes, NJ, USA). Peripheral blood mononuclear cells (PBMCs) were isolated by centrifugation through Ficoll-Paque (GE Healthcare, PA, USA). PBMCs were washed, counted, and stained before being used to isolate naïve and memory B cells as described previously (23). The naïve NGS sequences had average nucleotide mutation frequencies (MF) of 1.3% and average replacement mutation frequencies (RMF) of 1.4% for over 10,000 sequences, thus indicating low frequency of mutation errors due to PCR amplification and NGS sequencing. The memory NGS sequences had average MFs of 8.7% and average RMFs of 17.7% for roughly 3,700 sequences, which is similar to Sanger sequencing calculations (24). Previous work examining base-specific error rates identified a skewing toward the following order: A ≥ T > G > C (25) in sequences that had been PCR amplified prior to NGS. We also observe an overall increase in A and decrease in G mutations in the paired samples as expected from this earlier work, even though we used a different polymerase for PCR amplification (we used Phusion High-fidelity DNA polymerase from New England Biolabs, while Shao and colleagues used Hi Fidelity Platinum Taq from Invitrogen) (Figure S1 in Supplementary Material).

### Single B-cell receptor database generation using Sanger DNA sequencing

Sanger fourth family of variable heavy-chain region (*VH4*) sequence databases were generated at UTSWMC by nested PCR of single-sorted CD19+ CSF B cells using degenerate PCR primers, Taq DNA Polymerase (Promega, Madison, WI, USA), and Sanger DNA sequencing as previously described (12, 26, 27).

### PCR of antibody genes from CSF-derived B-cell pools

Details of this method are provided in the Supplementary Material. All PCR reactions were performed using Phusion High-fidelity DNA polymerase (New England Biolabs, Ipswich, MA, USA) to minimize amplification errors.

### Next-generation sequencing of CSF-derived B-cell pools

Details of this method are provided in the Supplementary Material. Sequencing was done on the 454 GS FLX DNA Sequencer using the 454 Titanium chemistry (Roche/454, Branford, CT, USA) according to the manufacturer’s recommended protocols.

### NGS 454 data processing

Details of this method are provided in the Supplementary Material. In total, we analyzed 212 Sanger-generated sequences from single B cells and 16,984 unique NGS-generated sequences. Sanger sequencing produced an average of 30 unique *VH4* sequences per patient, although fewer than 20 sequences were obtained from three of the patients (C1, C4, and C7) (Table 2). Although NGS sequencing produced an average of 2,426 unique *VH4* sequences per patient, fewer than 1,000 sequences were obtained from two of the patients (C3 and C4) and one of these patients only yielded 14 unique *VH4* sequences. The large number of unique sequences in the NGS database relative to the number of B cells in the cell pellet is a consequence of the accumulation of PCR- and NGS-generated errors in the sequence database. Our focus here is to examine how well the sequence characteristics of the original patient template pools are maintained through NGS sequencing by comparing the patient’s Sanger database.

**Table 2:** **Sequence database size summary**.

Patient ID	No. of Sanger *VH4* sequences	No. of B cells in cell pellet for NGS	No. of unique NGS *VH4* sequences
C1	7	29	2,475
C2	41	100	2,213
C3	61	100	14
C4	14	30	596
C5	25	100	5,020
C6	46	100	4,290
C7	18	100	2,376
Average	30		2,426

*For each patient, the initial *VH4* sequences obtained by Sanger sequencing of single B cells, the number of B cells in the cell pellet used for NGS PCR and sequencing, and the number of unique *VH4* NGS sequences after filtering are indicated. Of note, a typical Sanger-based antibody repertoire can take several months to generate, whereas NGS-based repertoires can take as little as 1 week*.

### Mutation analyses

Sequence and mutation information was available and calculated from Chothia codons 31–92 (28, 29). This region includes complementarity determining region (CDR) 1 through framework regions (FR) 3 as originally defined by Kabat (30). Analyses were done for both nucleotide mutation frequency (MF) and amino acid RMF. CDR and FR region mutation data were obtained by separating mutations in CDR1 and CDR2 from those in FR2 and FR3 and normalizing based on the lengths of the specific region.

At the codon level, mutations were characterized as either replacement or silent mutations (RM or SM) and R:S ratios were calculated as RM divided by SM. AGS scores were calculated as previously described: they are the sum for each AGS codon (31b; 40; 56; 57; 81; 89) of [RMF at the AGS codon minus the average RMF (1.6) in a healthy control peripheral blood database divided by the standard deviation (0.9) of the average RMF of the same healthy control database] (16). Patients with AGS scores above 6.8 are identified as “RRMS.”

### Statistical analyses

*VH4* and *JH* gene frequencies, mutated nucleotide frequencies, and AGS-contributing codon frequencies were grouped by platform and compared by Chi-squared analysis. MF, R:S ratios, and AGS scores were evaluated as patient-specific data points and their distributions between platforms were compared by Wilcoxon matched-pairs signed rank test. Statistical significance for all methods was attributed to *p*-values ≤0.05. Using the follow-up diagnosis as the basis for evaluation, specificity, sensitivity, and accuracy were calculated for OCB, Sanger AGS, and NGS AGS. Specificity was calculated as (no. of correct CIS assessments)/(no. of CIS samples); sensitivity was calculated as (no. of correct RRMS assessments)/(no. of RRMS samples); and accuracy was calculated as (no. of correct assessments)/(no. of samples).

## Results

Sanger sequencing has been the gold standard to define the antibody repertoires of patients with autoimmune diseases such as MS (26, 31–37). Such findings have provided necessary information to further our understanding on the role of B cells and their antibody products on the pathology of MS, the application of new targeting therapeutics, and the development of new diagnostic tools. NGS represents an advanced sequencing method to query even massive B-cell pools, and has already been applied to defining B-cell clonality in MS patients (22). However, it is critical to evaluate whether this new sequencing technology properly represents the unique features that were previously established by Sanger sequencing for antibody genetics in B cells from the CSF of MS patients.

**Figure 1:**
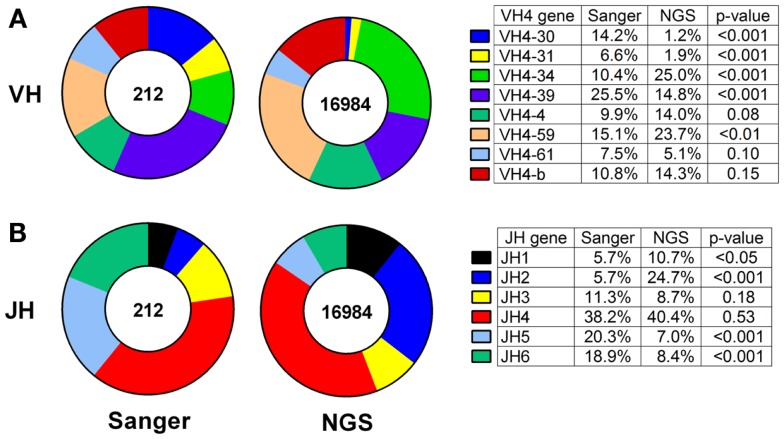
***VH4* gene distributions show cross-platform variation for samples from both patients with RRMS and CIS**. *VH4* **(A)** and *JH* **(B)** gene calls were obtained by IMGT alignment. Total sequences used in Sanger sequencing and next-generation sequencing (NGS) databases are indicated inside the pie charts. Statistically significant differences between the frequencies of individual genes were identified by Chi-squared test (*p*-value: N.S. ≥0.05).

Thus, we compared the antibody gene repertoires generated from single CSF-derived B cells using Sanger sequencing and those generated from CSF B-cell pools using NGS in a cohort of MS/CIS patients. There were significant differences in the frequency of individual VH4 gene usage between the platforms, although the relative abundance of individual *VH4* gene segments by rank was globally consistent (Figure 1A). In the comparison of the Sanger and NGS databases, *VH4-30*, *VH4-34*, and *VH4-39* sequences show significant differences in abundance. *VH4-39* was the most abundant gene segment in the Sanger database, but is the third most abundant gene segment in the NGS database. All the other *VH4* gene segments remain in the same ranked order of abundance in both databases. The rank order of the *VH4-b*, *VH4-4*, and *VH4-61* gene segments do not significantly vary between platforms. *VH4-59* has a significant increase in NGS (15–24%; *p* = 0.004), which does not alter its rank. One noticeable difference is the lower abundance of long *VH4* gene segments (*VH4-30*, *VH4-31*, *VH4-39*, and *VH4-61*) in the NGS database (23%) compared with the Sanger database (54%).

JH usage is important because skewing from the normal distribution of dominant JH4 usage (38) can be evidence of self-reactivity (39). *JH4* remained the most abundant gene segment in both the Sanger and NGS databases (compare 38–40%; *p* = 0.53) and *JH3* remained the fourth most abundant gene segment in both databases (compare 11–9%; *p* = 0.18) (Figure 1B). *JH5* and *JH6* were significantly decreased in the NGS database, whereas *JH1* and *JH2* were significantly increased and resulted in significant differences in frequencies of these four JH genes between the platforms.

**Figure 2:**
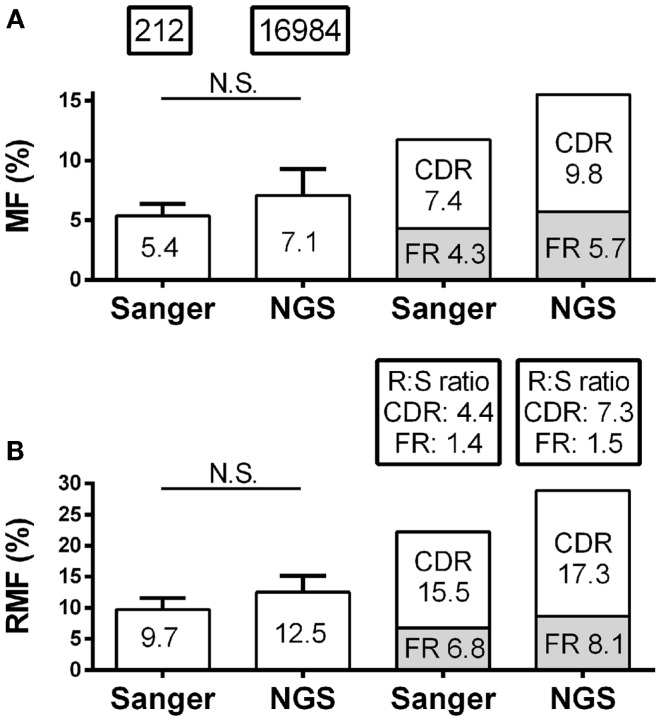
**Mutation characteristics of *VH4* sequences in RRMS and CIS patients are shown**. Sanger sequence data include 212 sequences with 2265 total point mutations and 1386 total replacement mutations (RM). Next-generation sequencing (NGS) data include 16,984 unique sequences with 263,764 total point mutations and 154,457 total replacement mutations (RM). **(A)** Mutation frequency (MF) analysis was done by nucleotide; **(B)** replacement mutation frequencies (RMF) analysis was done by codon. MF and RMF were calculated by patient, and bar graphs show mean (indicated on the bar graphs) and S.D. (statistical significance of the distributions was tested for by Wilcoxon matched-pairs signed rank test; N.S. ≥0.05). MF, RMF, and R:S ratios for CDR and FR regions were calculated independently by region for each patient and are shown as patient means.

Skewing of mutation frequency and/or placement of mutations in antibody genes from the CSF of MS patients is well established (12–14, 26). It is important, therefore, that the identification of the mutation accumulation and distribution is similar regardless of the platform by which it was generated. With regard to the accumulation of mutations, the overall nucleotide MF for individual patients by Sanger and NGS were similar (5.4–7.1%; *p* = 0.16) (Figure 2A; Table S1 in Supplementary Material). The RMF was also consistent between platforms (Figure 2B; Table S1 in Supplementary Material), again with a non-significant increase in NGS (9.7–12.5%; *p* = 0.11). With regard to the distribution of mutations, the MF and RMF were also appropriately highest in the CDRs, which are the antigen-contacting sites. The FRs, which are the structural support regions of the antibody genes, had relatively few MF and RMF accumulations as expected (Figures 2A,B). The replacement to silent mutation ratios (R:S ratios) in the CDR regions increase from patient to patient (average 4.4–7.3; *p* = 0.58) in the NGS platform, but without a significant trend emerging (Figure 2B). The R:S ratios in the FR regions were not significantly altered across platforms (1.4–1.5; *p* = 0.94).

**Figure 3:**
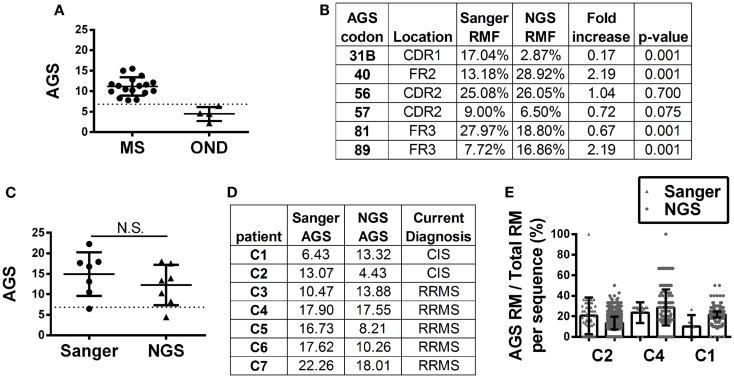
**Antibody gene signature (AGS) in RRMS and CIS patients is shown**. **(A)** Unpaired Sanger sequence datasets for multiple sclerosis (MS, includes relapsing-remitting, primary and secondary progressive MS samples) and other neurological disease (OND) cohorts. Each data point represents a single patient sequence pool that was not analyzed by NGS. The dotted line represents the AGS cut-off point of 6.8 above which patients are expected to convert to relapsing-remitting multiple sclerosis (RRMS). Mean and standard deviation are shown. **(B)** Replacement mutation frequencies (RMF) of each of the six AGS codons were calculated relative to the total AGS RM in each dataset. *P*-values were calculated by Chi-squared test. **(C)** Each data point represents a single patient sequence pool. The dotted line represents the AGS cut-off point of 6.8 above which patients are expected to convert to RRMS. Mean and standard deviation are shown. Statistical significance of the distributions was tested for by Wilcoxon matched-pairs signed rank test (N.S. ≥0.05). **(D)** The AGS scores of the seven paired patients are shown here. **(E)** The percent of total RMs that belong to the AGS pattern in each sequence was mapped for three patients with different types of AGS score shifts from one platform to another. The boxes indicate mean and the error bars S.D.

Antibody gene signature scoring by Sanger sequencing showed initial success on a pilot cohort in identifying MS patients or CIS patients who will convert to MS (16), which has been confirmed in larger sample cohorts (Figure 3A). To understand how antibody repertoire generation by NGS might affect AGS scoring calculations, we analyzed and compared the RMF at each codon position that defines the AGS (Figure 3B). Only codons 56 and 57 of the AGS maintained similar RMF to the Sanger repertoires. RMF at codons 40 and 89 were significantly increased and RMF at codons 31B and 81 were significantly decreased in comparison to the Sanger repertoires.

Despite these fluctuations in mutation distributions among the six AGS codons, we observed a non-significant change (14.9–12.2; *p* = 0.22) in the paired samples of the average AGS score with the NGS platform (Figure 3C) (16). Two patients who have not yet received a confirmed RRMS diagnosis (patients C1 and C2) did not have consistent AGS scores between the Sanger and NGS databases (Figure 3D). However, all of those patients who did have RRMS or converted to RRMS after sampling showed consistent classification of disease by both Sanger sequencing and NGS. In addition, the specificity (50%), sensitivity (100%), and accuracy (85.7%) of properly identifying patients that have MS or would convert to MS in the future in this small cohort was the same for NGS-based, Sanger-based, and OCB biological testing. However, the small size of the cohort precludes any conclusion regarding the utility of NGS-based AGS scoring as a viable diagnostic test.

Finally, to understand these fluctuations in AGS scores between the two platforms, we show the distribution of AGS codon RM frequency and how it affects AGS scores for three representative samples. For example, in the Sanger repertoire of patient C2, approximately 21% of all RMs are within the AGS codons (Figure 3E) resulting in an AGS score of 13.07. In the NGS repertoire of this same patient, only 14% of all RMs are within the AGS codons resulting in a decreased AGS score of 4.43. Conversely, the NGS repertoire of patient C1 had an increased AGS score compared to the Sanger repertoire because of an increased percentage of RMs in AGS codons relative to all codons (compare 15% in Sanger vs. 22% in NGS). Patient C4 had similar percentages of RM in AGS codons on both platforms (26 vs. 25%), and thus had similar AGS scores on both platforms (17.90 vs. 17.55%).

## Discussion

Radiological testing to support MS diagnosis has excelled and is indispensable in the diagnosis of MS, whereas development of biological tests to support MS diagnosis has been more challenging. One type of biological testing that is on the horizon is next-generation DNA sequencing (NGS), which can be used to query the antibody genetics of even massive B-cell pools (18–22). Historically, this technology has been very successful in tracking minimal residual disease in cancer patients (18). More recently, the power of this technology has been used to demonstrate that focused B-cell clones in the CSF of MS patients are identifiable in the vast peripheral B-cell pools of the same patients (22). Thus, the use of NGS to pursue biological questions in MS has become a reality.

Our goal for this study was to advance beyond clonality queries and address whether the features of antibody genetics that we had observed in CSF-derived B cells from MS patients with regard to antibody gene distribution (i.e., skewing toward *VH4* family usage) and somatic hypermutation accumulation (i.e., AGS) could be confirmed using this deeper sequencing method. This is important because NGS is now readily available commercially, and its possible limitations must be understood to best translate the information that we obtain from it. To do this, we compared paired antibody repertoires generated from single CSF-derived B cells using Sanger sequencing and antibody repertoires generated from CSF B-cell pools using NGS. This is the first time that there has been a direct comparison of this new technology to Sanger sequencing, which is the gold standard in the field.

Overall, we found that NGS and Sanger sequence data were similar with regard to general mutational profiles but differed somewhat in the distribution of *VH4* sub-family members recovered. Due to the similarity between the sequences of the *VH4* sub-family gene segments, the divergence in *VH4* distribution may be partially due to an increase in sequencing errors in the NGS database, the most common of which is insertion and deletion (indel) errors, particularly in regions that contain homopolymers or stretches containing two or more identical nucleotides (40). The reported frequency of indels generated by the Roche/454 platform is in the range of 3.8 to 5 × 10^−3^ (41, 42). Indels are easily detected by alignment of NGS-generated sequences to published *VH4* sequences using the IMGT/High V-Quest tool (43). Since we remove all non-productive (with stop codons or frameshift mutations) or misaligned ( <85% homology) antibody sequences, our NGS databases should contain very few sequences with indels. In order for a sequence with indels to pass our filters, they would have to contain multiple complementary indel events in close proximity – an extremely unlikely scenario. Nucleotide substitution errors can also occur (44), but we used a very high-fidelity DNA polymerase to generate our NGS-based antibody repertoires so that the MF between the Sanger and NGS databases would be similar.

All five patients who had or converted to RRMS were properly identified using our AGS biological test method by Sanger sequencing or NGS. There was some fluctuation in the AGS scores obtained for these paired samples between the two platforms, which could be due to a decreased representation in NGS of the long *VH4* genes that contain codon 31b. The AGS scoring system is based on MF at six codons, which includes 31b. Thus, if genes containing 31b are not properly represented in the NGS repertoire database in comparison to Sanger database, a decrease in AGS scores would be a natural consequence. Despite these differences in Sanger and NGS repertoire generation, identification of MS patients or CIS patients that would convert to MS remained the same between the two platforms.

The two CIS patients who did not convert to RRMS at follow-up are representative of biological testing complications due to patient care received. CIS patient C1 was at high risk to develop MS. The Sanger-based AGS score was below the 6.8 cut-off point, but the NGS-based AGS score was above the cut-off point suggesting that this patient would convert to RRMS in the future. CIS patient C2 was OCB positive at the time of sampling, with a single brain lesion noted by MRI, and was thus considered at low risk to develop MS. The Sanger-based AGS score was above the 6.8 cut-off point, but the NGS-based AGS score was below the cut-off point. In both of these cases, the patients were placed on disease modifying therapy shortly after sampling, making it difficult to determine what the natural progression of their demyelinating event may have been. Of note, patient C5 who was on steroids at the time of sampling and converted to RRMS had an AGS score above the 6.8 cut-off point by both platforms.

This study suggests that the transition from single B-cell Sanger sequencing to high-throughput NGS of pooled B cells is feasible with the application of appropriate sequence filtering methods to efficiently remove sequences containing errors generated during sample processing and sequencing. The implementation of appropriate quality metrics to identify and remove as many process-generated errors as possible will be critical for the successful use of NGS to better understand the antibody genetics of MS and for the future development of a clinically useful NGS diagnostic test based on the AGS scoring algorithm. These results will need to be confirmed in a larger cohort of patients using NGS-based antibody repertoire generation before consideration as a diagnostic tool can be made.

## Author Contributions

William H. Rounds participated in data processing and filtering, wrote the database comparison programs, performed the data analysis, and drafted the manuscript. Ann J. Ligocki collected the samples, generated the Sanger data, and participated in NGS database generation. Mikhail K. Levin carried out sequence alignment and processing. Benjamin M. Greenberg participated in patient recruitment, study design, and helped draft the manuscript. Douglas W. Bigwood participated in data processing and filtering, as well as data analysis and study design. Eric M. Eastman participated in study design, data analysis, and helped to draft the manuscript. Lindsay G. Cowell participated in study design, sequence alignment and processing, and data analysis. Nancy L. Monson conceived of and coordinated the study, participated in patient recruitment, study design, data analysis, and helped to draft the manuscript. All authors read and approved the final manuscript.

## Conflict of Interest Statement

William H. Rounds, Ann J. Ligocki, Mikhail K. Levin, Lindsay G. Cowell, and Nancy L. Monson have no competing interests. Douglas W. Bigwood and Eric M. Eastman are employees of DioGenix and, as such, they each own equity shares in the company and are inventors on pending patents relating to the work described in this publication. Benjamin M. Greenberg owns equity shares in DioGenix. This research was supported in part by a Sponsored Research Agreement that DioGenix has with UTSWMC.

## Supplementary Material

The Supplementary Material for this article can be found online at http://www.frontiersin.org/Journal/10.3389/fneur.2014.00166/abstract

## References

[1] Polman CH Reingold SC Banwell B Clanet M Cohen JA Filippi M Diagnostic criteria for multiple sclerosis: 2010 revisions to the McDonald criteria. Ann Neurol (2011) 69(2):292–302 10.1002/ana.22366 21387374 PMC3084507

[2] Milo R Miller A Revised diagnostic criteria of multiple sclerosis. Autoimmun Rev (2014) 13(4–5):518–24 10.1016/j.autrev.2014.01.012 24424194

[3] Frohman EM Havrdova E Lublin F Barkhof F Achiron A Sharief MK Most patients with multiple sclerosis or a clinically isolated demyelinating syndrome should be treated at the time of diagnosis. Arch Neurol (2006) 63(4):614–9 10.1001/archneur.63.4.614 16606781

[4] Reske D Petereit HF Heiss WD Difficulties in the differentiation of chronic inflammatory diseases of the central nervous system – value of cerebrospinal fluid analysis and immunological abnormalities in the diagnosis. Acta Neurol Scand (2005) 112(4):207–13 10.1111/j.1600-0404.2005.00414.x 16146488

[5] Tintore M Rovira A Rio J Tur C Pelayo R Nos C Do oligoclonal bands add information to MRI in first attacks of multiple sclerosis? Neurology (2008) 70(13 Pt 2):1079–83 10.1212/01.wnl.0000280576.73609.c6 17881717

[6] Petzold A Intrathecal oligoclonal IgG synthesis in multiple sclerosis. J Neuroimmunol (2013) 262(1–2):1–10 10.1016/j.jneuroim.2013.06.014 23890808

[7] Cepok S Rosche B Grummel V Vogel F Zhou D Sayn J Short-lived plasma blasts are the main B cell effector subset during the course of multiple sclerosis. Brain (2005) 128(Pt 7):1667–76 10.1093/brain/awh486 15800022

[8] Owens GP Bennett JL Gilden DH Burgoon MP The B cell response in multiple sclerosis. Neurol Res (2006) 28(3):236–44 10.1179/016164106X98099 16687047

[9] Antel J Bar-Or A Roles of immunoglobulins and B cells in multiple sclerosis: from pathogenesis to treatment. J Neuroimmunol (2006) 180(1):3–8 10.1016/j.jneuroim.2006.06.032 16934338

[10] Lucchinetti C Brück W Parisi J Scheithauer B Rodriguez M Lassmann H Heterogeneity of multiple sclerosis lesions: implications for the pathogenesis of demyelination. Ann Neurol (2000) 47(6):707–17 10.1002/1531-8249(200006)47:6<707::AID-ANA3>3.0.CO;2-Q 10852536

[11] Harp CT Ireland S Davis LS Remington G Cassidy B Cravens PD Memory B cells from a subset of treatment-naïve relapsing-remitting multiple sclerosis patients elicit CD4+ T-cell proliferation and IFN-γ production in response to myelin basic protein and myelin oligodendrocyte glycoprotein. Eur J Immunol (2010) 40(10):2942–56 10.1002/eji.201040516 20812237 PMC3072802

[12] Harp C Lee J Lambracht-Washington D Cameron E Olsen G Frohman E Cerebrospinal fluid B cells from multiple sclerosis patients are subject to normal germinal center selection. J Neuroimmunol (2007) 183(1–2):189–99 10.1016/j.jneuroim.2006.10.020 17169437 PMC2034205

[13] Owens GP Winges KM Ritchie AM Edwards S Burgoon MP Lehnhoff L VH4 gene segments dominate the intrathecal humoral immune response in multiple sclerosis. J Immunol (2007) 179(9):6343–51 10.4049/jimmunol.179.9.6343 17947712

[14] Bennett JL Haubold K Ritchie AM Edwards SJ Burgoon M Shearer AJ CSF IgG heavy-chain bias in patients at the time of a clinically isolated syndrome. J Neuroimmunol (2008) 199(1–2):126–32 10.1016/j.jneuroim.2008.04.031 18547652 PMC2572301

[15] Bennett JL Owens GP Cerebrospinal fluid proteomics: a new window for understanding human demyelinating disorders? Ann Neurol (2012) 71(5):587–8 10.1002/ana.23595 22522475

[16] Cameron EM Spencer S Lazarini J Harp CT Ward ES Burgoon M Potential of a unique antibody gene signature to predict conversion to clinically definite multiple sclerosis. J Neuroimmunol (2009) 213:123–30 10.1016/j.jneuroim.2009.05.014 19631394 PMC2785005

[17] Ligocki AJ Lovato L Xiang D Guidry P Scheuermann RH Willis SN A unique antibody gene signature is prevalent in the central nervous system of patients with multiple sclerosis. J Neuroimmunol (2010) 226(1–2):192–3 10.1016/j.jneuroim.2010.06.016 20655601 PMC2937103

[18] Boyd SD Marshall EL Merker JD Maniar JM Zhang LN Sahaf B Measurement and clinical monitoring of human lymphocyte clonality by massively parallel VDJ pyrosequencing. Sci Transl Med (2009) 1(12):12ra23 10.1126/scitranslmed.3000540 20161664 PMC2819115

[19] Boyd SD Gaeta BA Jackson KJ Fire AZ Marshall EL Merker JD Individual variation in the germline Ig gene repertoire inferred from variable region gene rearrangements. J Immunol (2010) 184(12):6986–92 10.4049/jimmunol.1000445 20495067 PMC4281569

[20] Arnaout R Lee W Cahill P Honan T Sparrow T Weiand M High-resolution description of antibody heavy-chain repertoires in humans. PLoS One (2011) 6(8):e22365 10.1371/journal.pone.0022365 21829618 PMC3150326

[21] Logan AC Gao H Wang C Sahaf B Jones CD Marshall EL High-throughput VDJ sequencing for quantification of minimal residual disease in chronic lymphocytic leukemia and immune reconstitution assessment. Proc Natl Acad Sci U S A (2011) 108(52):21194–9 10.1073/pnas.1118357109 22160699 PMC3248502

[22] von Budingen HC Kuo TC Sirota M van Belle CJ Apeltsin L Glanville J B cell exchange across the blood-brain barrier in multiple sclerosis. J Clin Invest (2012) 122(12):4533–43 10.1172/JCI63842 23160197 PMC3533544

[23] Ireland SJ Blazek M Harp CT Greenberg B Frohman EM Davis LS Antibody-independent B cell effector functions in relapsing remitting Multiple Sclerosis: clues to increased inflammatory and reduced regulatory B cell capacity. Autoimmunity (2012) 45(5):400–14 10.3109/08916934.2012.665529 22432732

[24] Ligocki AJ Rounds WH Cameron EM Harp CT Frohman EM Courtney AM Expansion of CD27 plasmablasts in transverse myelitis patients that utilize VH4 and JH6 genes and undergo extensive somatic hypermutation. Genes Immun (2013) 14(5):291–301 10.1038/gene.2013.18 23594958 PMC4326094

[25] Shao W Boltz VF Spindler JE Kearney MF Maldarelli F Mellors JW Analysis of 454 sequencing error rate, error sources, and artifact recombination for detection of low-frequency drug resistance mutations in HIV-1 DNA. Retrovirology (2013) 10:18 10.1186/1742-4690-10-18 23402264 PMC3599717

[26] Monson NL Brezinschek HP Brezinschek RI Mobley A Vaughan GK Frohman EM Receptor revision and atypical mutational characteristics in clonally expanded B cells from the cerebrospinal fluid of recently diagnosed multiple sclerosis patients. J Neuroimmunol (2005) 158(1–2):170–81 10.1016/j.jneuroim.2004.04.022 15589051

[27] Brezinschek HP Brezinschek RI Lipsky PE Analysis of the heavy chain repertoire of human peripheral B cells using single-cell polymerase chain reaction. J Immunol (1995) 155(1):190–202 7602095

[28] Chothia C Lesk AM Canonical structures for the hypervariable regions of immunoglobulins. J Mol Biol (1987) 196(4):901–17 10.1016/0022-2836(87)90412-8 3681981

[29] Al-Lazikani B Lesk AM Chothia C Standard conformations for the canonical structures of immunoglobulins. J Mol Biol (1997) 273(4):927–48 10.1006/jmbi.1997.1354 9367782

[30] Kabat EA Te Wu T Perry HM Gottesman KS Foeller C Sequences of Proteins of Immunological Interest. Darby, PA: Diane Publishing (1992).

[31] Qin Y Duquette P Zhang Y Talbot P Poole R Antel J Clonal expansion and somatic hypermutation of V(H) genes of B cells from cerebrospinal fluid in multiple sclerosis. J Clin Invest (1998) 102(5):1045–50 10.1172/JCI3568 9727074 PMC508971

[32] Colombo M Dono M Gazzola P Roncella S Valetto A Chiorazzi N Accumulation of clonally related B lymphocytes in the cerebrospinal fluid of multiple sclerosis patients. J Immunol (2000) 164(5):2782–9 10.4049/jimmunol.164.5.2782 10679121

[33] Owens GP Ritchie AM Burgoon MP Williamson RA Corboy JR Gilden DH Single-cell repertoire analysis demonstrates that clonal expansion is a prominent feature of the B cell response in multiple sclerosis cerebrospinal fluid. J Immunol (2003) 171(5):2725–33 10.4049/jimmunol.171.5.2725 12928426

[34] Qin Y Duquette P Zhang Y Olek M Da RR Richardson J Intrathecal B-cell clonal expansion, an early sign of humoral immunity, in the cerebrospinal fluid of patients with clinically isolated syndrome suggestive of multiple sclerosis. Lab Invest (2003) 83(7):1081–8 10.1097/01.LAB.0000077008.24259.0D 12861047

[35] Baranzini SE Jeong MC Butunoi C Murray RS Bernard CC Oksenberg JR B cell repertoire diversity and clonal expansion in multiple sclerosis brain lesions. J Immunol (1999) 163(9):5133–44 10528220

[36] Owens GP Kraus H Burgoon MP Smith-Jensen T Devlin ME Gilden DH Restricted use of VH4 germline segments in an acute multiple sclerosis brain. Ann Neurol (1998) 43(2):236–43 10.1002/ana.410430214 9485065

[37] Owens GP Burgoon MP Anthony J Kleinschmidt-DeMasters BK Gilden DH The immunoglobulin G heavy chain repertoire in multiple sclerosis plaques is distinct from the heavy chain repertoire in peripheral blood lymphocytes. Clin Immunol (2001) 98(2):258–63 10.1006/clim.2000.4967 11161983

[38] Briney BS Willis JR McKinney BA Crowe JE Jr High-throughput antibody sequencing reveals genetic evidence of global regulation of the naive and memory repertoires that extends across individuals. Genes Immun (2012) 13(6):469–73 10.1038/gene.2012.20 22622198

[39] Meffre E Chiorazzi M Nussenzweig MC Circulating human B cells that express surrogate light chains display a unique antibody repertoire. J Immunol (2001) 167(4):2151–6 10.4049/jimmunol.167.4.2151 11489999

[40] Bolotin DA Mamedov IZ Britanova OV Zvyagin IV Shagin D Ustyugova SV Next generation sequencing for TCR repertoire profiling: platform-specific features and correction algorithms. Eur J Immunol (2012) 42(11):3073–83 10.1002/eji.201242517 22806588

[41] Loman NJ Misra RV Dallman TJ Constantinidou C Gharbia SE Wain J Performance comparison of benchtop high-throughput sequencing platforms. Nat Biotechnol (2012) 30(5):434–9 10.1038/nbt.2198 22522955

[42] Georgiou G Ippolito GC Beausang J Busse CE Wardemann H Quake SR The promise and challenge of high-throughput sequencing of the antibody repertoire. Nat Biotechnol (2014) 32(2):158–68 10.1038/nbt.2782 24441474 PMC4113560

[43] Alamyar E Duroux P Lefranc MP Giudicelli V IMGT((R)) tools for the nucleotide analysis of immunoglobulin (IG) and T cell receptor (TR) V-(D)-J repertoires, polymorphisms, and IG mutations: IMGT/V-QUEST and IMGT/HighV-QUEST for NGS. Methods Mol Biol (2012) 882:569–604 10.1007/978-1-61779-842-9_32 22665256

[44] Kircher M Kelso J High-throughput DNA sequencing – concepts and limitations. Bioessays (2010) 32(6):524–36 10.1002/bies.200900181 20486139

