# Supplementary material for: The Antibody Genetics of Multiple Sclerosis: Comparing Next-Generation Sequencing to Sanger Sequencing
Source: Front Neurol. 2014 Sep 16;5:166. doi: 10.3389/fneur.2014.00166 (PMC4165282; doi:10.3389/fneur.2014.00166)
Supplement: Supplementary file 1 [file DataSheet_1.PDF]

Supplementary Material

The antibody genetics of multiple sclerosis: comparing next-generation sequencing to Sanger sequencing

William H. Rounds[1], Ann J. Ligocki[1], Mikhail K. Levin[2], Benjamin M. Greenberg[1], Douglas W. Bigwood[4], Eric M. Eastman[4], Lindsay G. Cowell[2], Nancy L. Monson[1, 3]*

[1] Department of Neurology and Neurotherapeutics, UT Southwestern Medical Center, Dallas, TX, United States.
[2] Department of Clinical Sciences, UT Southwestern Medical Center, Dallas, TX, United States.
[3] Department of Immunology, UT Southwestern Medical Center, Dallas, TX, United States.
[4] DioGenix Inc., Bethesda, MD, United States

*** Correspondence:** Dr. Nancy L. Monson, UT Southwestern Medical Center, Dept. of Neurology and Neurotherapeutics, 6000 Harry Hines Blvd., NL9.110E, Dallas, TX, 75390-8813, USA, nancy.monson@utsouthwestern.edu

1. Supplementary Methods
1.1. PCR of antibody genes from CSF-derived B cell pools

NGS *VH4* sequence databases were generated at SeqWright Genomic Services (Houston, TX) starting with cDNA generated at UTSWMC from pools of sorted B cells from the same patients who were analyzed using the single cell Sanger sequencing approach described above. cDNA was amplified using a Primer Extension Preamplification (PEP) protocol as previously described by our group (1) and modified from an earlier protocol (2). This template then underwent comprehensive PCR amplification of *VH4* sequences using a modified nested PCR strategy based on the methods described above, except that pools of non-degenerate primers replaced the degenerate primers and patient-specific barcode sequences were added using the 4-primer Amplicon Tagging strategy developed by Fluidigm (South San Francisco, CA) to allow for multiplex sequencing. Amplified *VH4* sequences for each patient and controls were tagged with a unique barcode sequence (see below). All PCR reactions were performed using Phusion High-fidelity DNA polymerase (New England Biolabs, Ipswich, MA) to minimize of amplification errors.

1.2. Next generation sequencing of CSF-derived B cell pools

Sets of nested external and internal forward primers were designed to anneal to sequences in the framework 1 (FR1) regions of all *VH4* family members. Sets of nested external and internal reverse primers were designed to anneal all *JH* members downstream of the conserved CTGGGG motif. To allow for the incorporation of specific barcode sequences to the amplicons generated for each patient, the 5’ ends of the forward internal primers were extended to include the common sequence 1 (CS1) tag and the 5’ ends of the reverse internal primers were extended to include the common sequence 2 (CS2) tag (Fluidigm). Patient-specific barcode sequences were added to nested PCR amplicons by performing a third PCR reaction using forward primers

that contain the 454A primer, 4 nucleotide key, unique 10 nucleotide MID barcode, and CS1' sequences, and reverse primers that contain the 454B primer, 4 nucleotide key, unique 10 nt MID barcode, and CS2' sequences. All *VH4* and *JH* custom primers were synthesized by Integrated DNA Technologies (Coralville, IA). The 454/barcode primers were purchased from Fluidigm.

Four external and four internal PCR reactions were performed for each sample to increase the total amount of patient DNA processed and minimize the chance of any stochastic effects for CSF samples that have very low numbers of *VH4*-expressing B cells (see Discussion). Each external PCR reaction consisted of 3.0 µL of PEP cDNA, 10.0 µL 2X Phusion DNA Polymerase Master mix (NEB), 1.0 µL each of 10 µM pooled external forward and reverse PCR primers and water to bring the total volume to 20 µL. PCR cycling conditions were as follows: 98°C for 3 minutes followed by 23 cycles of 98°C for 10 seconds, 68°C for 10 seconds, 72°C for 10 seconds. The last 72°C extension was extended to 10 minutes followed by a 4°C hold.

Each internal PCR reaction consisted of 3.0 µL DNA from the external PCR reaction, 10.0 µL 2X Phusion DNA Polymerase Master mix (NEB), 1.0 µL each of 10 µM pooled CS1/CS2-tagged internal forward and reverse PCR primers and water to bring the total volume to 20 µL. PCR cycling conditions were as follows: 98°C for 1 minute followed by 10 cycles of 98°C for 10 seconds, 68°C for 10 seconds, 72°C for 10 seconds then 21 cycles of 98°C for 10 seconds, 72°C for 10 seconds. The last 72°C extension was extended to 10 minutes followed by a 4°C hold.

An aliquot of each of the four internal PCR reactions for each patient was analyzed on a 2% agarose-TAE gel. Internal PCR reactions that yielded a visible band of the appropriate size (320-350 bp) were pooled for each patient. PCR amplicons of the appropriate size were gel purified using QIAquick Gel Extraction Kit (Qiagen, Valencia, CA). Patient-specific MID barcode sequences and the 454 primer sequences were then added in a single barcoding PCR reaction using 20 ng of gel-purified nested PCR product and the same conditions as for the external PCR reaction.

PCR amplicons of the appropriate size were gel purified from 2% agarose-TAE gels using the QIAquick Gel Extraction Kit (Qiagen, Valencia, CA) again after addition of the 454/barcode sequences. DNA was quantitated using PicoGreen and equimolar amounts of DNA from each clinical patient plus controls were pooled and sequenced together. Prior to emulsion PCR (emPCR), pooled DNA samples were analyzed using the 2100 Bioanalyzer (Agilent Technologies, Santa Clara, CA) to confirm that the DNA fragment sizes in the pool were of the appropriate length and that there was a minimal amount of short sequences, e.g. primers and primer dimer. The pooled DNA was then used for emPCR and sequenced on the 454 GS FLX DNA Sequencer using the 454 Titanium chemistry (Roche/454, Branford, CT) according to the manufacturer's recommended protocols.

1.3. NGS 454 data processing

Each unique sequence was aligned to germline gene segment sequences using the IMGT/HighV-QUEST tool (3). IMGT outputs were compiled using a Perl program developed at UTSWMC (1, 4). All subsequent data processing steps described here were performed using a

combination of Perl and SQL database programs developed in-house. Initial filtering removed any sequence which met at least one of the following criteria: out of frame, truncated read length, less than 85% homology to germline sequence, and alignment errors (as indicated by IMGT). Because the NGS sequences were generated from pools of 100 or fewer B cells per sample, we found certain sequences to be highly amplified. Combined with processing error rates (both PCR and 454 platform based), this results in some sequences that are found in multiple samples, which we termed sequence crossover.

Since CDR3 sequences of the *VH* chains should not match from sample to sample (5), we adopted a strategy to identify sequences in multiple samples that share the same CDR3 subsequence. In order to properly filter these out, we needed to remove crossover sequences with exact CDR3 sequence matches, but also include highly similar CDR3s which fall into 3 categories that are still in-frame: up to 3 single mismatches, a homopolymer insertion plus deletion, and 3 homopolymer insertions or deletions. By using Levenshtein distance comparison that measures the number of indels and mismatched nucleotides that separate each CDR3 nucleotide sequence pair (6, 7), we were able to cluster the crossover CDR3s using a maximal distance of 3, to account for the rare in-frame homopolymer indels. This matched previous work done on T cell receptor CDR3 sequences (8). Any sequence cluster present in multiple samples was removed from all sample sequence pools. The exceptions were clusters with ≥ 99% representation in a single sample, in which case we used this conservative cut-off to justify the sample source of the crossover sequence cluster, and only removed its members from the other sample databases.

2.1. Supplementary Table 1. Mutation characteristics of *VH4* sequences in RRMS and CIS patients. Mutation frequency (MF) analysis was done by nucleotide. Replacement mutation frequency (RMF) analysis was done by codon. MF and RMF means were calculated by patient and statistical significance of the frequency distributions between Sanger and NGS databases was tested for by Wilcoxon matched-pairs signed rank test be.

Patient ID	MF		RMF	
	Sanger	NGS	Sanger	NGS
C1	4.75%	7.73%	8.42%	13.84%
C2	6.61%	9.53%	12.38%	14.70%
C3	5.83%	4.93%	10.69%	9.28%
C4	6.42%	3.49%	11.43%	8.12%
C5	5.00%	8.47%	8.79%	14.64%
C6	3.74%	6.52%	7.18%	13.37%
C7	5.15%	8.80%	9.07%	13.63%
Wilcoxon test	0.156		0.109	

2.2. Supplementary Table 2. Additional patient sample summary. Includes unpaired samples only analyzed by Sanger sequencing as shown in Figure 3A. Initial diagnosis at the time of sample collection is indicated for each patient in the study. Abbreviations in table: OCB, oligoclonal bands; CIS, clinically isolated syndrome; RRMS, relapsing-remitting multiple

sclerosis; SPMS, secondary-progressive multiple sclerosis; PPMS, primary-progressive multiple sclerosis; ON, optic neuritis; CIDP, chronic inflammatory demyelinating polyneuropathy; PNS, paraneoplastic neurological syndrome.

Patient ID	Initial diagnosis[1]	OCB status	Follow-up diagnosis[2]	Follow-up time[3]	Age[4]	Gender
S1	Myelopathy	POS	RRMS	3	25	F
S2	CIS	POS	RRMS	18	23	F
S3	CIS	POS	RRMS	3	61	M
S4	RRMS	NA	NA	NA	33	F
S5	RRMS	NA	NA	NA	25	F
S6	SPMS	NA	NA	NA	42	F
S7	PPMS	NA	NA	NA	45	F
S8	RRMS	NA	NA	NA	34	F
S9	RRMS	NA	NA	NA	41	F
S10	SPMS	POS	NA	NA	39	F
S11	PPMS	POS	NA	NA	46	F
S12	RRMS	POS	NA	NA	42	F
S13	ON	POS	RRMS	2	23	F
S14	ON	POS	RRMS	3	32	M
S15	ON	POS	RRMS	5	27	F
S16	ON	POS	RRMS	5	49	F
S17	ON	POS	RRMS	3	35	F
S18	ON	NEG	NA	NA	39	M
S19	ON	POS	NA	NA	44	F
S20	NA	NA	CIDP	39	60	F
S21	PNS	NA	NA	NA	66	M
S22	ON	POS	NA	NA	40	F

[1] At time of sampling using 2005 McDonald Criteria
[2] Using 2010 McDonald Criteria
[3] Since sampling (months)
[4] At time of sampling (yrs)

Supplementary Figure 1

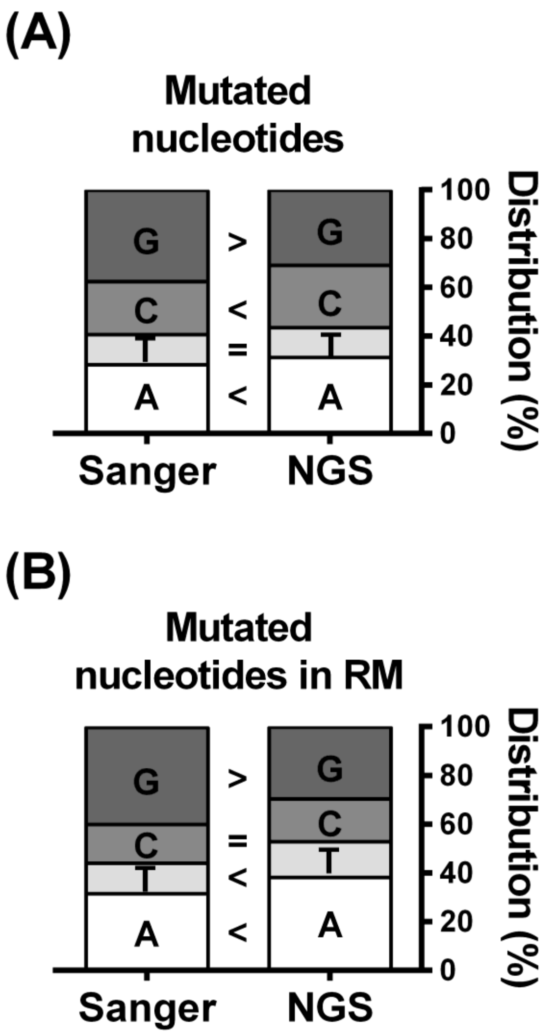

3. Supplementary Figure 1. Cross-platform comparison of germline nucleotide mutations in *VH4* sequences. Sanger sequence data includes 212 sequences with 2265 total point mutations and 1386 total replacement mutations (RM). Next-generation sequencing (NGS) data includes 16,984 unique sequences with 263,764 total point mutations and 154,457 total replacement mutations (RM). Germline nucleotides with any point mutations (A) or with point mutations that caused a replacement mutation (B) were evaluated for nucleotide frequency (> or < indicate significant and = indicates non-significant frequency changes between platforms, where significance required a p-value < 0.05). Statistically significant differences between the frequencies of mutated nucleotides were identified by Chi-squared test.

3. References

1. Ligocki AJ, Rounds WH, Cameron EM, Harp CT, Frohman EM, Courtney AM, et al. Expansion of CD27 plasmablasts in transverse myelitis patients that utilize VH4 and JH6 genes and undergo extensive somatic hypermutation. *Genes Immun* (2013). Epub 2013/04/19. doi: 10.1038/gene.2013.18
gene201318 [pii]. PubMed PMID: 23594958.
2. Tiller T, Meffre E, Yurasov S, Tsuiji M, Nussenzweig MC, Wardemann H. Efficient generation of monoclonal antibodies from single human B cells by single cell RT-PCR and expression vector cloning. *J Immunol Methods* (2008) **329**(1-2):112-24. Epub 2007/11/13. doi: S0022-1759(07)00312-2 [pii]
10.1016/j.jim.2007.09.017. PubMed PMID: 17996249; PubMed Central PMCID: PMC2243222.
3. Alamyar E, Duroux P, Lefranc MP, Giudicelli V. IMGT((R)) tools for the nucleotide analysis of immunoglobulin (IG) and T cell receptor (TR) V-(D)-J repertoires, polymorphisms, and IG mutations: IMGT/V-QUEST and IMGT/HighV-QUEST for NGS. *Methods Mol Biol* (2012) **882**:569-604. Epub 2012/06/06. doi: 10.1007/978-1-61779-842-9_32. PubMed PMID: 22665256.
4. Ligocki AJ, Lovato L, Xiang D, Guidry P, Scheuermann RH, Willis SN, et al. A unique antibody gene signature is prevalent in the central nervous system of patients with multiple sclerosis. *J Neuroimmunol* (2010) **226**(1-2):192-3. Epub 2010/07/27. doi: S0165-5728(10)00283-3 [pii]
10.1016/j.jneuroim.2010.06.016. PubMed PMID: 20655601.
5. Jackson KJ, Kidd MJ, Wang Y, Collins AM. The Shape of the Lymphocyte Receptor Repertoire: Lessons from the B Cell Receptor. *Front Immunol* (2013) **4**:263. Epub 2013/09/14. doi: 10.3389/fimmu.2013.00263. PubMed PMID: 24032032; PubMed Central PMCID: PMC3759170.
6. Levenshtein VI. Binary codes capable of correcting deletions, insertions, and reversals. *Soviet Physics Doklady* (1966).
7. Pieterse V, Black PE. Algorithms and Theory of Computation Handbook, CRC Press LLC, 1999, "Levenshtein distance". *Dictionary of Algorithms and Data Structures [online]* (eds. 22 August 2013; Available from: http://www.nist.gov/dads/HTML/Levenshtein.html).
8. Bolotin DA, Mamedov IZ, Britanova OV, Zvyagin IV, Shagin D, Ustyugova SV, et al. Next generation sequencing for TCR repertoire profiling: platform-specific features and correction algorithms. *Eur J Immunol* (2012) **42**(11):3073-83. Epub 2012/07/19. doi: 10.1002/eji.201242517. PubMed PMID: 22806588.
